# Exosomes from adipose-derived mesenchymal stem cells alleviate sepsis-induced lung injury in mice by inhibiting the secretion of IL-27 in macrophages

**DOI:** 10.1038/s41420-021-00785-6

**Published:** 2022-01-10

**Authors:** Xiaoyan Wang, Danyong Liu, XiHe Zhang, LiuMing Yang, Zhengyuan Xia, Quanfu Zhang

**Affiliations:** 1 Doctoral Scientific Research Center, Lianjiang People’s Hospital, Zhanjiang, Guangdong 524400 China; 2 Department of Anesthesiology, Affiliated Hospital of Guangdong Medical University, Zhanjiang, Guangdong 524001 China; 3 Department of Gastroenterology and Hepatology, People’s Hospital of Lianjiang, Zhanjiang, Guangdong 524400 China; 4 Department of Obstetrics, Shenzhen Baoan Women’s and Children’s Hospital, Jinan Univesity, Shenzhen, Guangdong 518102 China

**Keywords:** Chronic obstructive pulmonary disease, Mesenchymal stem cells

## Abstract

Acute lung injury (ALI) represents a frequent sepsis-induced inflammatory disorder. Mesenchymal stromal cells (MSCs) elicit anti-inflammatory effects in sepsis. This study investigated the mechanism of exosomes from adipose-derived MSCs (ADMSCs) in sepsis-induced ALI. The IL-27r^−/−^ (WSX-1 knockout) or wild-type mouse model of sepsis was established by cecal ligation and puncture (CLP). The model mice and lipopolysaccharide (LPS)-induced macrophages were treated with ADMSC-exosomes. The content of Dil-labeled exosomes in pulmonary macrophages, macrophages CD68^+^ F4/80^+^ in whole lung tissues, and IL-27 content in macrophages were detected. The mRNA expression and protein level of IL27 subunits P28 and EBI3 in lung tissue and the levels of IL-6, TNF-α, and IL-1β were measured. The pulmonary edema, tissue injury, and pulmonary vascular leakage were measured. In vitro, macrophages internalized ADMSC-exosomes, and ADMSC-exosomes inhibited IL-27 secretion in LPS-induced macrophages. In vivo, IL-27 knockout attenuated CLP-induced ALI. ADMSC-exosomes suppressed macrophage aggregation in lung tissues and inhibited IL-27 secretion. ADMSC-exosomes decreased the contents of IL-6, TNF-α, and IL-1β, reduced pulmonary edema and pulmonary vascular leakage, and improved the survival rate of mice. Injection of recombinant IL-27 reversed the protective effect of ADMSC-exosomes on sepsis mice. Collectively, ADMSC-exosomes inhibited IL-27 secretion in macrophages and alleviated sepsis-induced ALI in mice.

## Introduction

Sepsis is a fatal syndrome featured by abnormal host response to invasive pathogens, which involves hemodynamic changes and leads to a variety of life-threatening organ dysfunction [1]. The lung represents the foremost vulnerable organ during sepsis, and notably, acute respiratory distress syndrome (ARDS), a clinical term of acute lung injury (ALI), constitutes one of the most salient prognostic factors of death in sepsis [2]. The pathophysiology of sepsis-induced ALI has not been fully elucidated. Currently, antibiotics and supportive measures remain the available treatments for patients with sepsis-induced ALI, but these measures have limited effects on reducing the mortality of sepsis [3], highlighting the urgent need to develop innovative and potent therapies for sepsis-induced ALI.

In the process of sepsis-induced ALI, the activation of inflammatory pathways results in the destruction of alveolar epithelial cells, the enhancement of epithelial permeability, and the flow of edema fluid into the alveolar cavity [4]. Emerging evidence has indicated that the excessive secretion of inflammatory cytokines contributes to the occurrence and development of ALI/ARDS, and also, the degree and duration of the inflammatory response can eventually determine the prognosis of patients with sepsis-induced ALI/ARDS [5, 6]. Interleukin 27 (IL-27) is a member of the IL-12 cytokine family, which is primarily produced by dendritic cells and macrophages consisting of the subunit protein IL-27p28 and Epstein Bar virus-induced protein 3 (EBI3) [7]. Like many other cytokines, IL-27 bears pleiotropic property that can restrain or induce ongoing inflammatory response [8]. IL-27 has been identified to participate in the pathology of sepsis, and elevated IL-27 can be detected in the serum of septic patients early after the onset of sepsis, which is considered as a diagnostic biomarker of sepsis [9]. IL-27 neutralizing antibody therapy can reduce pulmonary inflammation and alleviate ALI caused by sepsis [8]. The existing findings suggest that IL-27 inhibition may become a prospective therapeutic target for patients with sepsis-induced ALI.

Mesenchymal stem cells (MSCs) are pluripotent progenitor cells with self-renewal ability [10], which can ameliorate the immune response and reduce the mortality of sepsis patients by decreasing pro-inflammatory and increasing anti-inflammatory cytokines [11]. Adipose-derived MSCs (ADMSCs) represent one of the most promising stem cells for the treatment of sepsis [12]. Therapeutically administration of ADMSCs alleviates liver injury, reduces systematic inflammation, and increases the survival rate of septic rats [13]. It is well established that ADMSCs release exosomes that mimic the effects of their parental cells to play a functional role [14]. Exosomes are extracellular membrane nanovesicles that can mediate the intercellular communication by transferring proteins or nucleic acids (DNA and RNA) into target cells, thereby changing the behavior of receptor cells [15]. ADMSC-derived exosomes have been proven to be one of the most critical paracrine factors implicated in the treatment of sepsis [16–18]. For example, ADMSC-derived exosomes mitigate overwhelming systemic inflammatory response and organ injury and thereby improve the outcome of rat with sepsis syndrome [17]. However, the precise mechanism underlying ADMSC-derived exosomes in sepsis-induced ALI is unclear. Therefore, this study sought to investigate the exact role and mechanism of ADMSC-derived exosomes in sepsis-induced ALI, which shall confer novel insights into the development of stem cell-based therapy in sepsis-induced ALI.

## Results

### Knockout of IL-27 attenuated lung injury induced by CLP

**Fig. 1:**
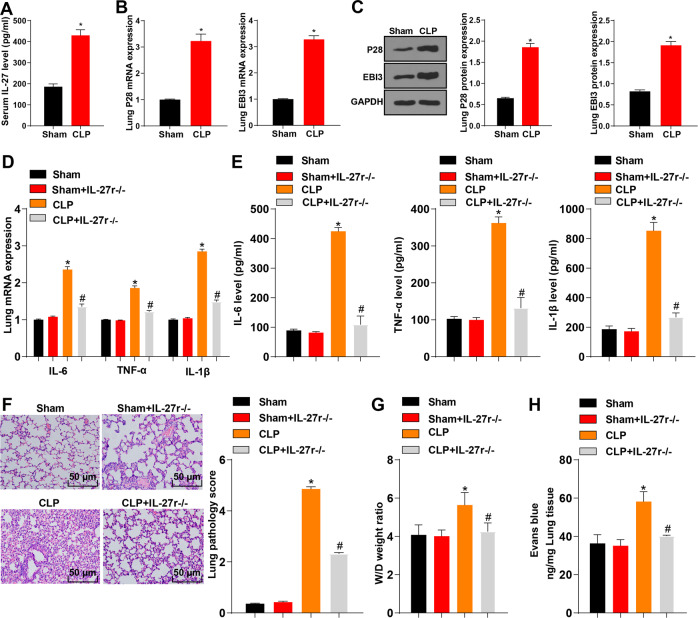
IL-27 was highly expressed in mice with CLP-induced acute lung injury. **A** The level of serum IL-27 of mice in the sham group and CLP group was detected using ELISA kit. **B** The mRNA expressions of EBI3 and P28 in mouse lung tissue were detected using RT-qPCR. **C** The protein levels of EBI3 and P28 in mouse lung tissue were detected using Western blot. **D** The mRNA expressions of IL-6, TNF-α, and IL-1β in mouse lung tissue were detected using RT-qPCR. **E** The protein contents of IL-6, TNF-α and IL-1β in lung tissues of mice in each group were detected using ELISA. **F** The lung injury was detected using HE staining, scale bar = 50 μm. **G** The wet/dry ratio was used to calculate the formation of pulmonary edema, *N* = 8. H: 24 h after CLP, Evans blue dye was injected to measure pulmonary vascular leakage, *N* = 8. Measurement data are depicted as mean ± SD. The *t* test was used for the comparisons between two groups. One-way ANOVA was employed for the comparisons among multiple groups, followed by Tukey’s multiple comparisons test. *p < 0.05 vs. Sham group; #*p* < 0.05 vs. CLP group.

To explore the relationship between IL-27 and CLP-induced acute lung injury, we first detected the level of IL-27 in serum using ELISA, and detected the levels of IL-27 subunits EBI3 and P28 in lung tissue using RT-qPCR and Western blot. Compared with the sham-operated mice, the CLP-induced mice showed an increased level of IL-27 in serum (Fig. 1A, *p* < 0.05), and upregulated mRNA expressions and protein levels of EBI3 and P28 in lung tissue (Fig. 1B–C, *p* < 0.05). Next, IL-27r^**−**/**−**^ mice were used to further verify the effect of IL-27 on lung injury after CLP. Compared with the sham-operated mice, the CLP-induced mice had increased mRNA expressions and protein levels of IL-6, TNF-α, and IL-1 β in lung tissue, while compared with the CLP-induced mice, the CLP + IL-27r^**−**/**−**^ mice showed decreased mRNA expressions and protein levels of IL-6, TNF-α, and IL-1β in lung tissue (Fig. 1D–E, *p* < 0.05). Additionally, compared with the sham-operated mice, the CLP-induced mice showed elevated lung pathology score (Fig. 1F, *p* < 0.05) and decreased pulmonary edema (Fig. 1G, *p* < 0.05) and pulmonary vascular leakage (Fig. 1H, *p* < 0.05), while the IL-27r^**−**/**−**^ mice exhibited an opposite trend compared with the CLP mice (Fig. 1F–H, *p* < 0.05). There were no significant differences between the sham group and the sham + IL-27r^**−**/**−**^ group (Fig. 1D–H, *p* > 0.05). The results indicated that IL-27 played a vital role in the aggravation of lung injury after CLP.

### Isolation and identification of ADMSCs and ADMSC-exosomes

**Fig. 2:**
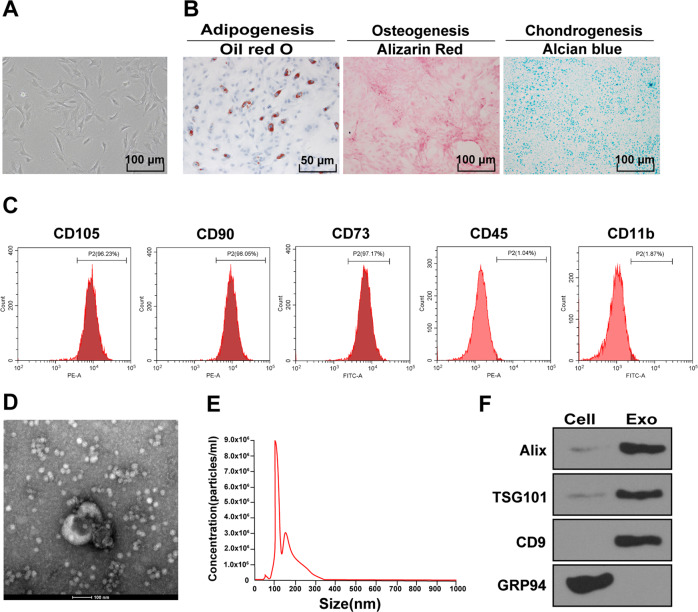
Isolation and identification of ADMSCs and ADMSC-exosomes. **A** The morphological characteristics of primary ADMSCs were observed under the inverted microscope. **B** Adipogenic (left), osteogenic (middle), and chondrogenic (right) differentiation experiments of ADMSCs. **C** The surface markers of ADMSCs were detected using flow cytometry. **D** The diameter of ADMSC-exosomes was observed under the TEM. **E** The diameter distribution and concentration of exosomes were detected using NTA. **F** The exosome marker proteins (Alix, CD9, and TSG101) and endoplasmic reticulum protein (GRP94) were detected using Western blot. The cell experiment was repeated three times independently.

ADMSCs and conditioned medium can attenuate sepsis injury in rats [19]. To explore whether ADMSCs can reduce sepsis injury through exosomes, we first isolated and purified ADMSCs from rat adipose tissue. Under the light microscope, ADMSCs were long fusiform or spindle shaped (Fig. 2A). Meanwhile, ADMSCs had good osteogenic, adipogenic, and chondrogenic differentiation ability (Fig. 2B). The surface markers of ADMSCs were detected using flow cytometry, and the results showed that the positive rates of CD73, CD90, and CD105 were over 95%, while the positive rates of CD45 and CD11b were lower than 2%, indicating that the extracted human ADMSCs were of high purity and could be used in subsequent experiments. ADMSC-exosomes were further isolated from the conditioned medium of ADMSCs, and measured using TEM and NTA. The results exhibited that the isolated ADMSC particles were vesicles with bilayer membrane in the range of 30–150 nm (Fig. 2D–E). Furthermore, Western blot results demonstrated that ALIX, TSG101, and CD9 could be detected in exosomes, but the non-exosome marker GRP94 was negative (Fig. 2F). All these results indicated that the ADMSC-exosomes were successfully isolated.

### ADMSC-exosomes inhibited the LPS-mediated release of IL-27 in macrophages

**Fig. 3:**
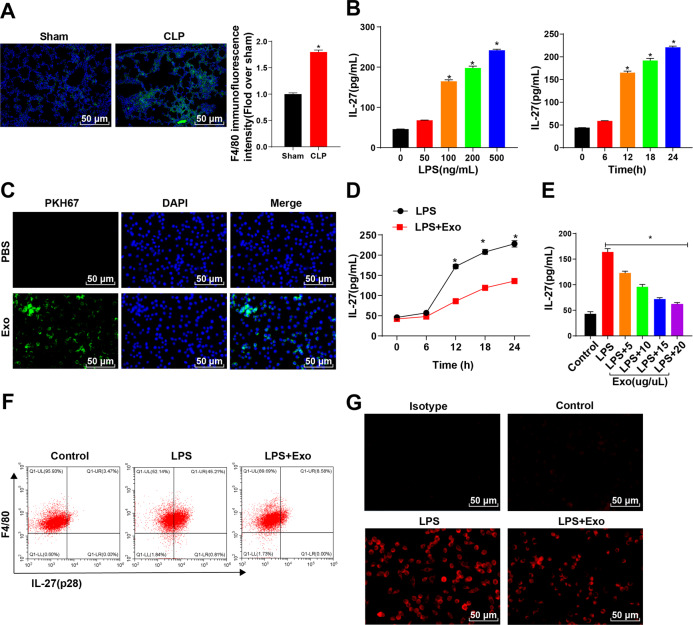
ADMSC-exosomes inhibited the LPS-mediated release of IL-27 in macrophages. **A** F4/80^+^ macrophages in lung tissue were detected using immunofluorescence (green staining) (nuclei were labeled by DAPI and presented blue staining). **B** The level of IL-27 in the supernatant of BMDMs treated with different concentrations of LPS for 12 h or LPS (100 ng/mL) for different times was detected using ELISA. **C** PKH67 (green)-labeled ADMSC-exosomes were co-cultured with BMDMs for 24 h, and then the fluorescence intensity of PKH67 in BMDMs was observed (DAPI-labeled nuclei were blue). **D** The time process of IL-27 release from BMDMs was detected using ELISA after the incubation of LPS (100 ng/mL) and ADMSC-exosomes (10 μg/μL). **E** The dose response of ADMSC-exosomes at different concentrations to IL-27 released by LPS-activated BMDMs within 12 h was detected using ELISA; Control indicated that the BMDMs were at rest. **F** BMDMs after LPS incubation were detected, with the addition of ADMSC-exosomes for 12 h or not. **G** The red staining of IL-27 in BMDMs was detected after 12 h-incubation, and Isotype represented the negative control. The cell experiment was repeated 3 times independently. Measurement data are depicted as mean ± SD. One-way ANOVA was employed for the comparisons among multiple groups, followed by Tukey’s multiple comparisons test. *p < 0.05.

Macrophages are an important source of IL-27 during sepsis [20]. Our study also found a significant enrichment of macrophages in the lung tissue of CLP mice (Fig. 3A). LPS was used to stimulate macrophages in an inflammatory state. The results showed that with the increase of LPS concentration and treatment time, the level of IL-27 in macrophage supernatant was increased gradually (Fig. 3B). We further explored the effect of ADMSC-exosomes on macrophages. ADMSC-exosomes were labeled with PKH67-green and it was found that macrophages could phagocytize a large number of exosomes (Fig. 3C). Additionally, ADMSC-exosomes was added to LPS-stimulated BMDMs and it was found that ADMSC-exosomes exerted a long-term inhibitory effect on the release of IL-27 in the supernatant of LPS-activated BMDMs (Fig. 3D). Moreover, ADMSC-exosomes effectively inhibited the release of IL-27 in macrophages in a dose-dependent manner (Fig. 3E). Next, we used flow cytometry was used to detect the level of IL-27 in BMDMs and the results indicated that the ratio of F4/80^+^ IL-27 ^+^BMDMs was notably decreased after ADMSC-exosomes incubation compared with LPS treatment alone (Fig. 3F). Immunofluorescence staining also showed that ADMSC-exosomes significantly reduced IL-27 level in BMDMs (Fig. 3G). Taken together, ADMSC-exosomes repressed the synthesis and secretion of IL-27 in macrophages.

### ADMSC-exosomes reduced the number of pulmonary macrophages and the release of IL-27 in sepsis mice

**Fig. 4:**
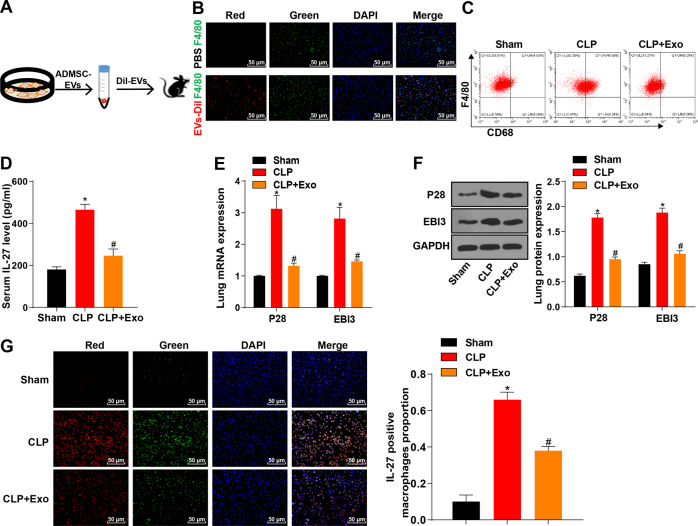
ADMSC-exosomes reduced the number of pulmonary macrophages and the release of IL-27 in sepsis mice. **A** ADMSC-exosomes were isolated, purified and labeled with Dil dye; flow chart of injection in CLP sepsis mice. **B** The co-localization of exosomes (Dil pre-labeled red) and macrophages (F4/80, green) in lung tissue was detected using immunofluorescence staining, scale bar = 25 μm. **C** The number of macrophages CD68^+^ F4/80 ^+^ in single-cell suspension of whole lung tissue was detected using flow cytometry. **D** The content of IL-27 in serum of CLP mice treated with ADMSC-exosomes was detected using ELISA. **E** The mRNA expressions of P28 and EB13 in lung tissue of CLP mice treated with ADMSC-exosomes were detected using RT-qPCR. **F** The protein levels of P28 and EB13 in lung tissue of CLP mice treated with ADMSC-exosomes were detected using Western blot. **G** The content of IL-27 in macrophages after the treatment of ADMSC-exosomes or not detected using immunofluorescence. Blue fluorescence indicated DAPI, green fluorescence indicated F4/80, and red fluorescence indicated IL-27. *N* = 8. Measurement data are depicted as mean ± SD. One-way ANOVA was employed for the comparisons among multiple groups, followed by Tukey’s multiple comparisons test. *p < 0.05 *vs*. Sham group; #*p* < 0.05 *vs*. CLP group.

Next, we further confirmed the role of ADMSC-exosomes in the mouse model of sepsis. Dil dye (red staining) was used to label exosomes (Fig. 4A). ADMSC-exosomes or PBS were intravenously injected into sepsis mice 4 h after CLP. First, we could observe the exosomes labeled by Dil dye (red staining) 6 h after injection. Macrophages were labeled with F4/80 (green), and it was found that exosomes and macrophages were accumulated in the lung tissue cells of CLP-induced sepsis mice (Fig. 4B). The number of macrophages (CD68^+^ F4/80^+^) in lung tissue of CLP-induced sepsis mice was notably increased, and ADMSC-exosomes could effectively reduce the number of macrophages (CD68^+^ F4/80^+^) (Fig. 4C). Additionally, ELISA elicited that the content of IL-27 in serum of CLP mice treated with ADMSC-exosomes was significantly reduced (Fig. 4D). RT-qPCR and Western blot results showed that EBI3 and P28 mRNA expressions and protein levels in lung tissue of CLP mice treated with ADMSC-exosomes were notably decreased (Fig. 4E–F). Furthermore, IL-27 level in lung macrophages was detected using immunofluorescence, and the results showed that ADMSC-exosomes treatment reduced IL-27 level in lung macrophages of CLP mice (Fig. 4G). Briefly, ADMSC-exosomes inhibited the aggregation of pulmonary macrophages and suppressed the release of IL-27.

### ADMSC-exosomes alleviated sepsis-induced lung injury by limiting the secretion of IL-27

**Fig. 5:**
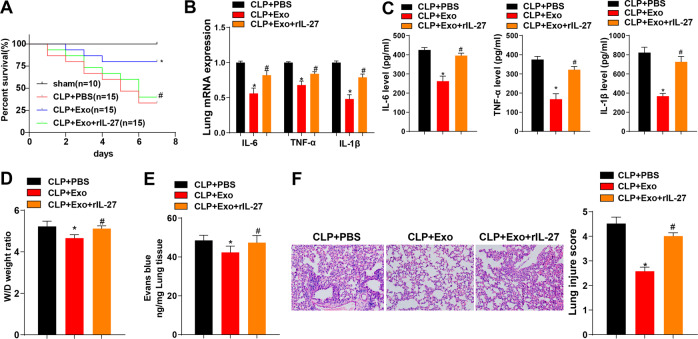
ADMSC-exosomes alleviated sepsis-induced lung injury by mediating the secretion of IL-17. **A** The survival rate of mice within 7 days (sham group: *N* = 10; other groups: *N* = 15). **B** The mRNA expressions of IL-6, TNF-α, and IL-1β in mouse lung tissue were detected using RT-qPCR 24 h after CLP induction, *N* = 8. **C** The protein contents of IL-6, TNF-α and IL-1β in mouse lung tissues were detected using ELISA. **D** The wet/dry ratio was used to calculate the formation of pulmonary edema, *N* = 8. **E** 24 h after CLP, Evans blue dye was injected to measure pulmonary vascular leakage, *N* = 8. **F** The morphological changes of lung were measured (scale bar = 25 μm) and the tissue injury was quantitatively analyzed (*N* = 8) using HE staining. Measurement data are depicted as mean ± SD. One-way ANOVA was employed for the comparisons among multiple groups, followed by Tukey’s multiple comparisons test. *p < 0.05 *vs*. CLP + PBS group; #*p* < 0.05 *vs*. CLP + Exo group. Kaplan-Meier method was used to calculate the survival rate. Log-rank test was used for univariate analysis.

We further explored the protective effect of ADMSC-exosomes on sepsis-induced lung injury. First, we measured the survival rate of mice within 7 days. Compared with that of the sham group, the survival rate of the CLP group was significantly decreased, and injection of ADMSC-exosomes notably elevated the survival rate of CLP mice. However, compared with that of the CLP + Exo group, the survival rate of the CLP + Exo + rIL-27 group was decreased (Fig. 5A). The cytokines and chemokines in mouse lung tissues were detected using RT-qPCR and ELISA. Compared with the CLP group, the CLP + Exo group had decreased contents of IL-6, TNF-α, and IL-1β in lung tissue of mice (Fig. 5B, C), and reduced pulmonary edema (Fig. 5D) and pulmonary vascular leakage (Fig. 5E). Histological staining showed that the lung injury of mice injected with ADMSC-exosomes was reduced (Fig. 5F). However, compared with those of the CLP + Exo group, the contents of IL-6, TNF-α, and IL-1β of the CLP + Exo + rIL-27 group were significantly elevated, accompanied by enhanced pulmonary edema, pulmonary vascular leakage, and lung tissue injury (Fig. 5B–F). Altogether, ADMSC-exosomes retarded sepsis-induced lung injury by limiting the secretion of IL-27.

## Discussion

**Fig. 6:**
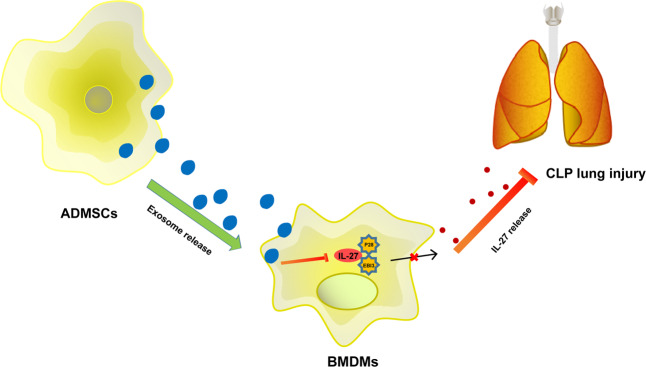
Mechanism diagram. ADMSC-exosomes inhibited IL-27 secretion in macrophages, thereby delaying sepsis-induced lung injury in mice.

Sepsis represents the foremost contributor to hospital deaths and poses heavy medical burden worldwide [21]. Recent evidence has indicated the crucial role of IL-27 in the pathogenesis of sepsis, and blocking IL-27 may be an alternative therapy for sepsis [22]. ADMSCs exhibit favorable therapeutic effects in preclinical models of sepsis and sepsis-induced ALI [23]. This study elucidated that ADMSC-exosomes protected mice against sepsis-induced ALI by inhibiting the secretion of IL-27 in macrophages (Fig. 6).

Pro-inflammatory and anti-inflammatory cytokines are released shortly after the onset of sepsis and continue to release throughout the course of the disease [24]. IL-27 emerges as a pro-inflammatory factor and mediates a variety of pro-inflammatory biological activities implicated in the pathogenesis of inflammation-related diseases, including sepsis [25]. In the current study, we established the mouse model of sepsis induced by CLP and then detected the level of IL-27 in serum of model mice, as well as the levels of IL-27 subunits (EBI3 and P28) in lung tissue. Our results showed that CLP-induced mice had an increased level of IL-27 in serum and upregulated mRNA expressions and protein levels of EBI3 and P28 in lung tissue. IL-27 expression in patients with sepsis and septic mice is notably increased and closely related to the severity and mortality of sepsis [25]. Moreover, CLP-induced mice showed increased pro-inflammatory cytokines such as IL-6, TNF-α, and IL-1β, elevated histological score of lung injury, and enhanced pulmonary edema and pulmonary vascular leakage. However, IL-27r^**−**/**−**^ mice exhibited an opposite trend compared with the CLP mice. Cao et al. also demonstrated that compared with wild-type mice, IL-27r^**−**/**−**^ mice have higher resistance to secondary bacterial pneumonia in sepsis environment, significantly lower bacterial burdens in lung and blood, and higher survival rate [26]. These findings suggested that IL-27 played a vital role in the aggravation of lung injury following sepsis.

Macrophages are momentous innate immune cells, which have a major part in immune homeostasis and inflammation owing to their ubiquitous existence and immunomodulatory functions [2]. The overwhelming inflammatory response is thought to play a key role in the host response to sepsis challenges, and such excessive inflammatory response relates to the accumulation of macrophages [25]. Consistently, our results showed a significant enrichment of macrophages in the lung tissue of CLP mice. Importantly, the release of IL-27 mainly occurs in mononuclear phagocytes (such as macrophages) and directly responds to various immune signals [27]. Hence, we used LPS to stimulate macrophages and found that with the increase of LPS concentration and the extension of treatment time, the level of IL-27 in macrophage supernatant was increased gradually.

ADMSC-exosomes can effectively protect sepsis-caused organ injury mainly by modulating the inflammatory-oxidative signaling axis [16]. Notably, emerging evidence has also clarified that the paracrine factors derived from ADMSCs can regulate immune system via interacting with multiple immune cells, including macrophages [28]. Therefore, we isolated and identified ADMSC-exosomes and further explored the effect of ADMSC-exosomes on macrophages. Our results demonstrated that macrophages could phagocytize a large number of exosomes and ADMSC-exosomes effectively suppressed the release of IL-27 in LPS-induced macrophages in a concentration-dependent manner. The biological functions of MSCs are associated with their immunomodulatory capacity, which is partly mediated by regulating IL-10 and IL-27 [29]. Extracellular vesicles are also demonstrated to restrain the expression of inflammatory cytokines including IL-27 in macrophages and delays autoimmune type 1 diabetes in mice [30]). Briefly, ADMSC-exosomes repressed the synthesis and secretion of IL-27 in macrophages. Subsequently, we further determined the role of ADMSC-exosomes in septic mice. It was found that exosomes and macrophages were accumulated in the lung epithelial cells of CLP mice. The number of macrophages in lung tissue of CLP mice was notably increased, and ADMSC-exosomes could effectively reduce the number of macrophages. The content of IL-27 in serum and lung macrophages of CLP mice treated with ADMSC-exosomes was significantly reduced. Altogether, in vivo experiments confirmed that ADMSC-exosomes suppressed the aggregation of pulmonary macrophages and repressed the release of IL-27. Furthermore, injection of ADMSC-exosomes notably increased the survival rate of CLP mice, decreased the levels of IL-6, TNF-α, and IL-1β in lung tissue, and reduced lung injury in CLP mice, while injection of recombinant-IL27 reversed the protective effect of ADMSC-exosomes on septic mice. The role of ADMSC-exosomes in repressing inflammatory/immune responses, abating lung/kidney injury, and decreasing mortality of septic rats has been revealed [17]. Inhibition of IL-27 contributes to reducing pulmonary inflammation and lung tissue injury, as well as improving the survival of mice in response to CLP [8]. Briefly, we were the first to demonstrate that ADMSC-exosomes retarded sepsis-induced ALI by mediating the secretion of IL-27.

To sum up, IL-27 level was increased in septic mice, and ADMSC-exosomes reduced the number of pulmonary macrophages and the release of IL-27, thus delaying sepsis-induced ALI in mice. This fundamental information might hint potential stem cell therapy for sepsis in the clinical trial. However, it is not clear whether ADMSC-exosomes can play a role in sepsis-induced ALI by carrying certain miRNAs. Also, the downstream signaling pathway of ADMSC-exosomes is unclear. In the future, we will focus on the possible downstream mechanism of ADMSC-exosomes in sepsis-induced ALI and conduct more prospective trials on the feasibility and safety of ADMSC-exosomes in the treatment of sepsis.

## Materials and methods

### Ethics statement

This study was performed following the approval from the Ethical Committee of Guangdong Medical University. All the animal experiments were implemented on the guide for the care and use of laboratory animals.

### Experimental animal

Male C57BL/6 mice aged 6–8 weeks were provided by Guangdong Medical Laboratory Animal Center, and IL-27r^**−**/**−**^ (WSX-1 knockout) C57BL/6 J mice were purchased from Jackson Laboratory (Bar Harbor, ME, USA). All mice were raised in a specific pathogen-free grade animal room at 20–22 °C with 40–60% humidity and maintained in a 12 h light/dark cycle. The mice had free access to food and water. The mice used in the experiments were kept in the animal room for at least one week.

### Isolation and identification of ADMSCs

Under aseptic conditions, adipose tissue samples were collected from the inguinal fat pad of 10 wild-type C57BL/6 mice asphyxiated by CO_2_. The adipose tissue was cut into small pieces and detached in 4 mg of 0.1% type I collagenase solution (Invitrogen Inc., Carlsbad, CA, USA; Gibco, Grand Island, NY, USA) at 37 °C for 15 min. The detached mixture was diluted with 4 mL Dulbecco’s modified Eagle’s medium (DMEM) containing 15% fetal bovine serum (FBS) and centrifuged at 1200 g for 15 min to separate cell particles from adipocytes. The supernatant was treated, and the cell particles were filtered through 200 μm nylon mesh to remove undigested tissue. The cell particles were incubated in DMEM-HG containing 15% FBS (Gibco), 100 U/mL penicillin, and 100 μg/mL streptomycin at 37 °C with 5% CO_2_. The medium was first replaced about 2 days after the start of culture. Thereafter, the medium was refreshed every 48 or 72 h. When the confluence reached 80–90%, ADMSCs were incubated with 0.05% trypsin (Sigma-Aldrich, Merck KGaA, Darmstadt, Germany) and 0.02% ethylene diamine tetraacetic acid (EDTA) until the second generation.

### Identification of multidirectional differentiation potential of ADMSCs

The isolated ADMSCs were cultured in DMEM-F12 (Hyclone, Logan, UT, USA) containing 10% FBS (10099141, Gibco) and 0.2% penicillin-streptomycin (Hyclone). The cells were passaged every three days. The ADMSCs at 3rd to 7th passage were used for following experiments. Then, ADMSCs were cultured in the OriCell™ osteogenic, adipogenic, or chondrogenic media (all from Cyagen, Guangzhou, China) in line with the manufacture instructions. The intracellular lipid droplets, calcium deposition, and proteoglycan accumulation were evaluated using 0.5% oil red O, 2% alizarin red, or 1% alcian blue staining, respectively.

### Flow cytometry

The ADMSCs at 3^rd^ passage with about 80% confluence were selected for surface identification. The medium was discarded and the cells were detached and centrifuged. After centrifugation, the precipitate was washed twice with phosphate-buffered saline (PBS) buffer, and the cells were counted. The cells were adjusted to 1 × 10^6^ cells/mL and transferred into a 15 mL centrifuge tube supplemented with 100 μL PBS buffer containing 2% FBS. Next, the cells were incubated with specific fluorescent flow cytometry antibodies CD90, CD105, CD73, CD45, and CD11b (1:100, FITC-labeled, BD Biosciences, San Jose, CA, USA, rat antimouse) at 4 °C for 30 min in the dark. The cells were resuspended with 3 mL PBS buffer, centrifuged and then added with 300 μL PBS buffer. In the control group, homomonoclonal antibody was used to determine the background marker as a gating strategy. The forward and side scatter methods were used to exclude cell aggregation and fragmentation analysis. The fluorescent cells were analyzed using the flow cytometer (BD FACSVerse). The positive rate of surface antigen was calculated using the FlowJo software (FlowJo, LLC, Ashland, OR, USA) and expressed as %.

The detection of bone marrow-derived macrophages (BMDMs) was consistent with that of ADMSCs. The surface antibodies IL-27 and F4/80 were from BD Biosciences (San Jose, CA, USA).

The lung tissues of mice were detached with collagenase and sieved with 70 μm nylon mesh to obtain single-cell suspension. The suspension was incubated with surface antibodies F4/80 and CD68 (BD Biosciences) respectively, fixed with 1% paraformaldehyde overnight, and detected using the flow cytometer on the next day.

### Isolation and incubation of BMDMs

BMDMs were isolated from the bone marrow of 6–8 week old male C57BL/6 mice (*N* = 5). The femur and tibia were separated and the adherent tissues were removed. An incision was made at both ends of the bone. The bone marrow was washed with DMEM containing 20% FBS, 100 μg/mL penicillin, 10 μg/mL streptomycin, and 30% L929 culture supernatant (containing macrophage colony stimulating factor; M-CSF) and cultured in a culture flask for 7 days.

### Drug treatment of macrophages

BMDMs were induced with lipopolysaccharide (LPS) of different concentrations (0, 50, 100, 200, and 500 ng/mL) for 12 h, or with 100 ng/mL LPS for different times (0, 6, 12, 18, and 24 h). In the subsequent exosome experiment, BMDMs were incubated with LPS (100 ng/mL; Sigma-Aldrich) for 12 h. Then, macrophages were treated with different concentrations of exosomes (0, 5, 10, 15, and 20 μg/μL) for 12 h, or with 10 μg/μL exosomes for different times (0, 6, 12, 18, and 24 h) [31, 32].

### Isolation and purification of ADMSC-exosomes

The medium/serum was centrifuged at 100000 g and 4 °C overnight to remove the exosomes in the serum. ADMSCs were cultured in conditioned medium (DMEM + 10% exosome-free serum) for 48 h. The medium was centrifuged at 300 g and 4 °C for 10 min to remove cell debris, at 2000 g for 20 min to remove cell debris or apoptotic bodies, and at 10,000 g for 40 min to remove large vesicles. After filtration with 0.22 μm filter, the sample was centrifuged at 11,0000 g and 4 °C for 70 min and then resuspended in PBS, followed by ultracentrifugation under the same conditions and resuspension in 100 μL sterile PBS. Thereafter, the iodixanol method was used for ultracentrifugation and further purification. The ultracentrifuged exosomes in the above steps were placed in12 layers of OptiPrep (Sigma-Aldrich) gradient consisting of 30, 27.5, 25, 22.5, 20, 17.5, 15, 12.5, 10, 7.5, 5, and 2.5% iodixanol in 20 mM Hepes (pH 7.2), 150 mM NaCl, 1 mM Na3VO4, and 50 mM NaF, then centrifuged with SW40 Ti rotor (Beckman Coulter, Chaska, MN, USA) at 110,000 g and 4 °C for 16 h, collected with 12 × 1 mL, washed with PBS, and centrifuged at 110,000 g and 4 °C for 70 min.

### Identification of exosomes

For NanoSight nanoparticle size analysis, 20 μg exosomes were dissolved in 1 mL PBS and whirled for 1 min to maintain the uniform distribution of exosomes. Then, the particle size distribution of exosomes was directly observed using the NanoSight nanoparticle tracking analyzer (NTA; Malvern, UK).

For transmission electron microscopy (TEM), 20 μL centrifuged exosome samples were loaded into the carbon-coated copper electron microscope grids for 2 min and negatively stained with phosphotungstic acid (12501-23-4, Sigma-Aldrich) for 5 min. The grids were then washed three times with PBS to remove the excess phosphotungstic acid solution and kept semi-dry with filter paper. The images were observed under TEM (H7650, Hitachi, Tokyo, Japan) at 80 kV.

The surface markers of exosomes were identified using Western blot. The exosome suspension was concentrated and the total protein concentration was determined using the bicinchoninic acid (BCA) kit (23227, Thermo Fisher Scientific Inc., Waltham, MA, USA). SDS-PAGE gel was prepared, and the proteins were denatured and subjected to electrophoresis. Afterward, the proteins were transferred to the membranes and the levels of the specific marker proteins of exosomes, including TSG101 (ab30871, Abcam, Cambridge, MA, USA), CD9 (ab92726, Abcam), Alix (ab76608, Abcam), and GRP94 (ab3674, Abcam) were detected.

### Uptake of exosomes

The purified ADMSC-exosomes were labeled with PKH67-green fluorescent kit (Sigma-Aldrich). Exosomes were resuspended in 1 mL Diluent C solution, and 4 μL PKH67 ethanol dye solution was added to 1 mL Diluent C to prepare 4 × 10^−6^ M dye solution. Then, 1 mL exosome suspension was mixed with the dye solution for 5 min and incubated with 2 mL 1% exosome-free FBS for 1 min to terminate the staining. The labeled exosomes were ultracentrifuged at 100,000 g for 2 h. The exosomes in the sample were enriched in the sucrose at the density of 1.13–1.19 g/mL, and then the exosomes were collected [33]. The PKH67-labeled exosomes were incubated with macrophages at 37 °C for 12 h. The cells were fixed with 4% paraformaldehyde and washed with PBS. The nuclei were stained with 4’,6-diamidino-2-phenylindole (DAPI) (Sigma-Aldrich).

### The mouse model of cecal ligation and puncture (CLP)-induced sepsis

Male wild-type C57BL/6 mice and IL27r^−/−^C57BL/6 mice (6–8 weeks old, 20–25 g) were used to establish the mouse model of CLP-induced sepsis. The cecum was ligated with 5-0 silk thread at the colon junction without breaking the continuity of the cecum, and then punctured twice with a 22 G needle. All animals were resuscitated by subcutaneous injection of saline. The sham-operation group (sham) did not perform cecum ligation and puncture, and other operations were the same as those of the CLP Group. All mice were anesthetized by intraperitoneal injection of 1% (50 mg/kg) pentobarbital sodium before operation.

### Survival analysis

Except the sham-operated mice (*N* = 10), CLP-induced sepsis mice were randomly assigned into three groups (*N* = 15/group): CLP + PBS group (wild-type C57BL/6 mice were subjected to CLP treatment and injected with PBS via tail vein), CLP + Exo group (wild-type C57BL/6 mice were subjected to CLP treatment and injected with ADMSC-exosomes via tail vein), and CLP + Exo + recombinant-IL27 (rIL27) group (wild-type C57BL/6 mice were injected with recombinant-IL27, subjected to CLP treatment, and injected with ADMSC-exosomes via tail vein). Recombinant-IL27 (1 μg; ProSpec-Tany TechnoGene Ltd, Ness-Ziona, Israel) was intraperitoneally injected into mice 2 h before CLP. ADMSC-exosomes (DiI labeled in vivo) (2 μg/kg body weight) were intravenously injected into mice 4 h after CLP. Mice received subcutaneous injection of imipenem (25 mg/kg) at 6, 24, and 48 h after CLP. The survival rate of mice was monitored within 7 days.

### Collection of plasma and tissue samples

In addition to survival analysis, CLP-induced sepsis mice were re-assigned into the sham group (sham-operated wild-type C57BL/6 mice), CLP group (wild-type C57BL/6 mice were treated with CLP operation), CLP + IL27r^**−**/**−**^ group (IL27r^**−**/**−**^ C57BL/6 mice were treated with CLP operation), CLP + PBS group (wild-type C57BL/6 mice were treated with CLP and injected with PBS via tail vein), CLP + Exo group (wild-type C57BL/6 mice were treated with CLP and injected with ADMSC-exosomes via tail vein), and CLP + Exo + rIL27 group (wild-type C57BL/6 mice were injected with recombinant-IL27, subjected to CLP treatment, and injected with ADMSC-exosomes via tail vein), with 16 mice in each group. Recombinant-IL27 (1 μg; ProSpec-Tany TechnoGene Ltd) was intraperitoneally injected into mice 2 h before CLP. ADMSC-exosomes (DiI labeled in vivo) (2 μg/kg body weight) were intravenously injected into mice 4 h after CLP. After 24 h of the CLP treatment, eight mice in each group were randomly selected for Evans blue dye assay 24 h after CLP to quantify vascular leakage in lung tissues, and the remaining eight mice were used for other studies.

The mice were anesthetized at 24 h after CLP, and then the blood was collected from the heart of the mice using a 1 mL syringe and transferred to the tube containing EDTA (BD Vacutainer, Franklin Lakes, NJ, USA). Plasma was centrifuged at 1600 g for 30 min and stored at −80 °C for subsequent analysis.

Then the lung tissues of mice in each group were isolated. The upper lobe of the left lung was collected and made into lung tissue suspension, and the wet weight of the lower lobe of the left lung was measured. The lower lobe of the right lung was subjected to hematoxylin and eosin (HE) staining; the middle lobe of the right lung was detected using immunofluorescence; the upper lobe of the right lung was stored at −80 °C for Western blot and reverse transcription-quantitative polymerase chain reaction (RT-qPCR).

### Enzyme-linked immunosorbent assay (ELISA)

The level of IL-27 in plasma and the levels of IL-6, TNF-α, and IL-1β in lung tissue suspension were detected using ELISA kits (R&D Systems, Minneapolis, MN, USA).

### Lung wet/dry ratio

The wet weight of the left lung of mice in each group was measured. Then the wet lung was dried at 60 °C for 48 h, and the dry weight was recorded. The pulmonary water content was calculated as the ratio of wet weight to dry weight.

### Measurement of pulmonary vascular leakage

Eight mice were randomly selected from each group. Evans blue dye assay was used to quantify the vascular leakage in lung tissue. Mice were injected with normal saline containing 1% Evans blue dye solution (Sigma-Aldrich) via tail vein. After 40 min, the mice were killed. Cardiopulmonary perfusion was performed and lung tissues were collected. The lung was weighed and placed in 1 mL formamide (Avantor, Center Valley, PA, USA) at 60 °C for 24 h to extract Evans blue dye. The sample was centrifuged at 626 g for 10 min to collect the supernatant. The concentration of Evans blue dye in the supernatant was quantified by measuring the absorbance at 620 nm, and calculated by the microplate reader according to the standard curve.

### Localization of exosomes in vivo

DiI label (C1036, Beyotime, Shanghai, China) was used for in vivo and in vitro exosome tracing. In short, the exosomes were resuspended in sterile PBS and incubated with 5 μM DiI for 10 min. Then, DiI-labeled exosomes were resuspended in sterile PBS for three times to remove DiI free and lipoproteins.

Lung tissues were collected 20 h after injection of DiI-labeled exosomes. Immunofluorescence staining was used to determine the distribution of exosomes and their location with macrophages. The exosomes were incubated with macrophage marker antibody F4/80 (sc-377009, 1:100, Santa Cruz Biotechnology, Santa Cruz, CA, USA) at 4 °C overnight. Next, the samples were incubated with goat antimouse IgG H&L (Alexa Fluor® 488) (ab150113, 1:500, Abcam) at room temperature for 2 h and stained with DAPI (Bioworld Technology, Shanghai, China). In each step, the samples were washed with PBS containing 10% bovine serum albumin and 0.25% Triton X-100. Finally, the images were captured using the fluorescence microscope (Eclipse Ti U, Nikon, Tokyo, Japan) and digital camera (DS Ri1, Nikon).

### Histological analysis

Lung tissues were collected from mice in each living group 24 h after CLP operation, fixed in 10% formalin buffer, embedded in paraffin, and cut into 5 um sections. The histological sections were stained with HE and the morphological damages were observed under the microscope. At least 10 fields were randomly selected in each mouse. Lung injury was scored according to alveolar congestion, hemorrhage, infiltration of neutrophils into air or vascular wall, and thickness of alveolar wall/hyaline plasma membrane [34]. The specific criteria are shown in Table 1. The lung injury was evaluated and scored by pathologists who did not know about the experimental grouping.

**Table 1:** Lung injury score parameters.

Changes of lung tissue structure	Index
None	1
Focal interstitial congestion and inflammatory cell infiltration <50%	2
Diffuse interstitial congestion and inflammatory cell infiltration >50%	3
Focal consolidation and inflammatory cell infiltration <50%	4
Diffuse consolidation and inflammatory cell infiltration >50%	5

### RT-qPCR

Total RNA was extracted from tissues and cells using the TRIzol reagent (16096020, Thermo Fisher Scientific). Then, 5 μg RNA was reverse transcribed into cDNA using the reverse transcription kit (K1622, Fermentas Inc., Ontario, CA, USA). SYBR Premix Ex Taq kit (Takara, Kyoto, Japan) and ABI StepOne real-time PCR system (Applied Biosystems, Inc., Carlsbad, CA, USA) were used for real-time PCR of mRNA. The relative expressions of genes were calculated using the 2^ΔΔCT^ method with GAPDH as an internal reference. The primer sequences are shown in Table 2.

**Table 2:** Primer sequences for RT-qPCR.

Gene		Primer sequence (5’-3’)
EBI3	F: GCTGCTCTTCCTGTCACTTGCC	R: TGAAGGACGTGGATCTGGTGGAG
P28	F: CTGCTTCCTCGCTACCACACT	R: CTCTTCCTCCTTGTCCTCCTCCTC
GAPDH	F: GGTGAAGGTCGGTGTGAACG	R: CTCGCTCCTGGAAGATGGTG
IL-6	F: ACTTCCATCCAGTTGCCTTCTTGG	R: TTAAGCCTCCGACTTGTGAAGTGG
TNF-α	F: GCGACGTGGAACTGGCAGAAG	R: GCCACAAGCAGGAATGAGAAGAGG
IL-1β	F: TCGCAGCAGCACATCAACAAGAG	R: TGCTCATGTCCTCATCCTGGA AGG

### Western blot

The cell proteins were collected and then lysed using lysis buffer (phosphatase inhibitor, protease inhibitor, and phenylmethylsulfonyl fluoride). The protein quantification was performed using the BCA method (Thermo Fisher Scientific). The proteins (10–20 μg) were separated with 8–12% acrylamide-Bis gel and transferred onto the polyvinylidene difluoride membranes (Merck Millipore, Billerica, MA, USA) with the pore size of 0.22 μm. The membranes were blocked with 5% skim milk for 1 h and cultured with the primary antibodies GAPDH (ab8245, Abcam), EBI3 (ab118500, Abcam), and P28 (ab118910, Abcam) overnight. On the next day, the membranes were cultured with the secondary antibody peroxidase-conjugated affinipure goat anti-rabbit IgG H&L (#111035003, Jackson ImmunoResearch) for 1 h and then visualized using the enhanced chemiluminescence reagent (Thermo Fisher Scientific). The protein blotting was analyzed using the Image J software (NIH Image, Bethesda, MD, USA). The relative protein content was expressed by the gray value of the corresponding protein band/the gray value of GAPDH protein band. The experiment was repeated 3 times.

### Immunofluorescence

Lung macrophages were assessed using immunofluorescence method. The frozen lung sections were retrieved with PBS, and then the cell membrane was penetrated with 0.3% Triton X-100 (50 μL) for 15 min. After that, the sections were placed in sodium citrate solution, heated for antigen recovery and blocked with normal goat serum. Then, the primary antibody F4/80 (sc-377009, 1:100, Santa Cruz) was added to the sections and incubated overnight in a humid chamber at 4 °C. The next day, the sections were maintained at room temperature for 1 h, washed with PBS, and then incubated with the secondary antibody goat antimouse IgG H&L (Alexa Fluor® 488) (ab150113, 1:500, Abcam) at room temperature for 1 h. Next, the sections were rinsed with TBST for three times, added with autofluorescence quencher for 5 min, and rinsed with tap water for 10 min. The nuclei were then stained with DAPI. The sections were washed 3 times, sealed, and observed under the fluorescence microscope. The five high-power visual fields were randomly selected for imaging, and the fluorescence intensity was quantitatively analyzed using the Image-Pro Plus 6.0 software (Media Cybernetics, Silver Spring, USA). The average value was calculated and normalized with sham as the control. The experiment was repeated three times.

IL-27 expression in lung macrophages was measured using immunofluorescence method. The specific steps were the same as above. Macrophages were identified using primary antibody F4/80 and secondary antibody. The expression of IL-27 was detected using the primary antibody IL-27 (sc-390482, 1:100, Santa Cruz) and secondary antibody goat antimouse IgG H&L (Alexa Fluor® 488) (ab150116, 1:200, Abcam). The expression of IL-27 in F4/80-positive cells was analyzed under the fluorescence microscope. The total numbers of F4/80-positive cells and IL-27-positive cells were counted using the ImageJ software, and the proportion of IL-27-positive cells in the total number of F4/80-positive cells was calculated. The average result of 5 fields was the final result of each section. The experiments were repeated three times.

### Statistical analysis

Statistical analyses were performed using the SPSS 21.0 (IBM Corp. Armonk, USA) and GraphPad Prism 6.0 (GraphPad Software Inc., San Diego, CA, USA). Measurement data are depicted as mean ± standard deviation (SD). The unpaired *t* test was used for the comparisons between two groups. One-way analysis of variance (ANOVA) was employed for the comparisons among multiple groups, followed by Tukey’s multiple comparisons test. The survival rate was calculated using Kaplan-Meier method and the survival difference analysis was carried out using log rank test. A value of *p* < 0.05 was regarded statistically significant.

## Supplementary information

Author Contribution Statement

## Data Availability

The data that support the findings of this study are available from the corresponding author upon reasonable request.
