# Supplementary material for: Exosomes from adipose-derived mesenchymal stem cells alleviate sepsis-induced lung injury in mice by inhibiting the secretion of IL-27 in macrophages
Source: Cell Death Discov. 2022 Jan 10;8:18. doi: 10.1038/s41420-021-00785-6 (PMC8744023; doi:10.1038/s41420-021-00785-6)
Supplement: Supplementary file 1 — Author Contribution Statement [file 41420_2021_785_MOESM1_ESM.docx]

**Author contributions**

XW, DL contributed to the study concepts, study design, and definition of intellectual content; XZ, LY contributed to the literature research; XW, DL contributed to the manuscript preparation and QZ contributed to the manuscript editing and review; XW, QZ, ZX contributed to the experimental studies and data acquisition; DL, XZ contributed to the data analysis and statistical analysis. All authors read and approved the final manuscript.
